# Oxygen-tolerant nitrogen fixation in a marine alga-colonizing *Planctomycetota*

**DOI:** 10.1128/aem.01316-25

**Published:** 2025-10-20

**Authors:** Zenghu Zhang, Ziwei Wang, Peichen Teng, Tong Yu, Yongyu Zhang

**Affiliations:** 1 School of Environmental and Municipal Engineering, Qingdao University of Technology 85437 , Qingdao, China; 2 Qingdao New Energy Shandong Laboratory, Qingdao Institute of Bioenergy and Bioprocess Technology, Chinese Academy of Sciences 66300, Qingdao, China; 3 Southern Marine Science and Engineering Guangdong Laboratory (Zhuhai) 590852 , Zhuhai, China; 4 Laboratory for Marine Biology and Biotechnology, Qingdao Marine Science and Technology Center 554912 , Qingdao, China; Georgia Institute of Technology, Atlanta, Georgia, USA

**Keywords:** *Planctomycetota*, algae-bacteria interaction, oxygen stress, nitrogen fixation, marine ecosystem

## Abstract

The microbiomes colonizing macroalgal surfaces orchestrate nutrient fluxes and symbiotic interactions within the algal environment. Among these communities, *Planctomycetota* are often dominant taxa. Although nitrogenase (*nif*) gene clusters have been identified in *Planctomycetota* isolates and metagenome-assembled genomes, functional validation of nitrogen fixation in pure culture has remained elusive. Moreover, the mechanisms enabling these bacteria to overcome oxygen sensitivity and fix nitrogen in algal-associated oxic niches remain unexplored. Here, we isolated *Crateriforma* sp. HD03, a *Planctomycetota* strain from the surface of *Saccharina japonica* (kelp), and provided the first experimental evidence of nitrogen-fixing activity in pure-cultured *Planctomycetota*. Strain HD03 harbors a complete *nifHDKBEN* gene cluster and exhibits a remarkable nitrogen fixation rate of 14.2 ± 1.5 nmol C_2_H_4_/(10⁷ cells)/h under aerobic conditions. Genomic and physiological analysis reveals a suite of adaptations that likely mitigate oxygen stress, including genes associated with biofilm formation, hopanoid lipid synthesis, FeSII protein, hydrogenase, and bacterial microcompartments. Notably, while strain HD03 demonstrates oxygen-tolerant nitrogen fixation in pure culture, co-culture experiments with kelp under a photoperiod revealed that *nifH* (nitrogenase reductase gene) expression peaks during the low-oxygen dark phase, indicating that HD03 utilizes diurnal rhythms to temporally separate nitrogen fixation from photosynthetic oxygen production. A genomic survey of 142 *Planctomycetota* strains from NCBI GenBank database and HD03 identified two distinct clades harboring complete *nifHDK* gene clusters, suggesting a nitrogen-fixing potential across the phylum. By bridging the gap between genomic potential and functional validation, this study establishes *Planctomycetota* as important but underappreciated contributors to marine nitrogen input.

**IMPORTANCE:**

*Planctomycetota* are abundant colonizers of macroalgal surfaces, yet their role in nitrogen fixation has remained unresolved despite genomic evidence of nitrogenase (nif) genes. Until now, no functional validation of nitrogen fixation in pure-cultured *Planctomycetota* has been reported. Here, we isolated *Crateriforma* sp. HD03 from kelp and for the first time demonstrated its ability to fix nitrogen in pure culture, confirming this key metabolic potential in marine *Planctomycetota*. Strain HD03 overcomes oxygen stress through a combination of biofilm formation and diurnal regulation of nifH expression, allowing nitrogen fixation under aerobic conditions to cope with the algal environment’s oxic nature. Furthermore, genomic surveys revealed nitrogen fixation gene clusters across multiple *Planctomycetota* clades, suggesting widespread nitrogen-fixing capability in this phylum. Collectively, these findings identify *Planctomycetota* as important nitrogen providers in the ocean.

## INTRODUCTION

The surface of macroalgae, such as kelp, represents a microecosystem critical to marine biogeochemical cycles, hosting specialized microbial communities that govern algal fitness and nutrient flux (1–3). Termed the “macroalgal holobiont,” these assemblages are shaped by photosynthesis-driven oxygen dynamics, algal exudates, and structural niches (4, 5), fostering intimate symbiotic relationships that influence carbon processing, stress resilience, and nitrogen availability (6–8). Among the dominant bacterial phyla, *Planctomycetota* stands out (9). For instance, *Planctomycetota* often dominate the bacterial community in the surface of brown algae, with a relative abundance exceeding 50%. Timo et al. found that the relative abundance of *Planctomycetota* in the attached bacterial communities of both juvenile and mature seagrasses was over 80%, significantly higher than in the surrounding water (<2%), with *Blastopirellula* and *Rhodopirellula* being the predominant genera (10). Similarly, Wiegand reported that over 70% of the bacterial community on the surface of brown algae in Australia belonged to *Planctomycetota* (11). *Planctomycetota* contribute to polysaccharide degradation and sulfur metabolism in the macroalgal environment (12). Yet, their role in nitrogen cycling—particularly nitrogen fixation—has remained enigmatic despite genomic hints.

Genomic and metagenomic analyses have identified nitrogenase (*nif*) gene clusters in *Planctomycetota*, including cultured *Blastopirellula* and *Crateriforma* strains, as well as metagenome-assembled genomes (MAGs) from oceanic samples (13–19). Environmental studies have further shown that *Planctomycetota* can comprise 4–20% of the diazotrophic community within bacterial assemblages harboring nitrogenase reductase gene (*nifH*) genes (20, 21). However, functional validation of nitrogen fixation in pure-cultured *Planctomycetota* has been absent, leaving uncertainty about whether these genes encode active enzymes or evolutionary remnants. This gap is particularly striking given the nitrogenase’s extreme sensitivity to oxygen, especially when contrasted with the dynamically oxic macroalgal environment generated by photosynthesis (22–25).

While heterotrophic nitrogen fixers in other systems employ strategies like biofilm-mediated microoxic niches or diurnal activity rhythms to mitigate oxygen stress, whether *Planctomycetota* deploy similar adaptations remains unknown. For example, particle-associated bacteria limit oxygen diffusion to protect nitrogenase (26, 27), and free-living strains form aggregates to sustain activity under aerobic conditions (28, 29). Cyanobacteria like *Crocosphaera* temporalize nitrogen fixation to dark periods to avoid oxygen produced during photosynthesis (30, 31). These analogies highlight potential mechanisms, but do not resolve the specific case of *Planctomycetota*.

Here, we address this knowledge gap using *Crateriforma* sp. HD03, a *Planctomycetota* strain isolated from kelp (*Saccharina japonica*). We provide the first experimental evidence of nitrogen fixation in *Planctomycetota*, demonstrating activity under aerobic conditions. This study bridges the genomic potential of *Planctomycetota* with their empirical validation in nitrogen fixation.

## RESULTS AND DISCUSSION

### Isolation and functional characterization of a nitrogen-fixing *Planctomycetota* strain from kelp surface

To address the gap in functional validation of nitrogen fixation in *Planctomycetota*, we cultured the surface microbiota of the brown alga *Saccharina japonica* using M14 medium (13) supplemented with 200 mg/L ampicillin, 1,000 mg/L streptomycin, and 20 mg/L cycloheximide. This selective strategy suppressed fast-growing bacteria and fungi, enabling the enrichment of slow-growing *Planctomycetota*. Serial streaking yielded pure culture HD03, whose 16S rRNA gene sequence showed 100% identity to *Crateriforma conspicua* Mal65^T^, a member of the family *Pirellulaceae* within the phylum *Planctomycetota*. However, digital DNA-DNA hybridization revealed a similarity of 50.9% (well below the 70% threshold for bacterial species), confirming HD03 as a genetically distinct strain.

**Fig 1:**
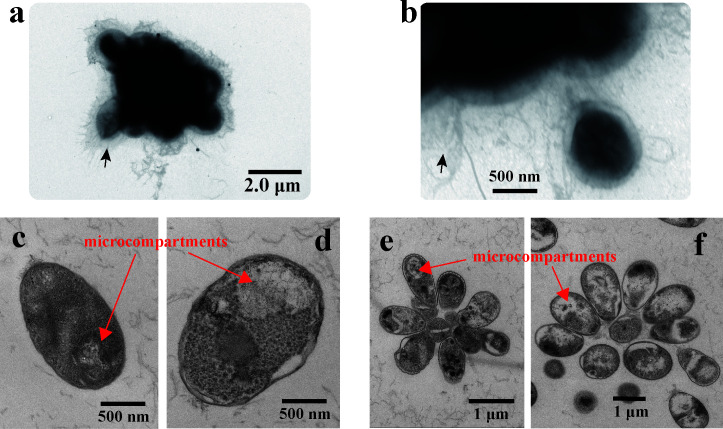
Transmission electron microscopy (TEM) of *Crateriforma* sp. HD03. (**a and b**) Unsectioned HD03 grown in M14 medium: (**a**) overview of cell aggregates and (**b**) magnified view of filamentous appendages (arrows). (**c–f**) Sectioned HD03: (**c**) single cell in nitrogen-containing M14 medium; (**d**) single cell in nitrogen-free NF medium; (**e**) cell aggregate in nitrogen-containing M14 medium; and (**f**) cell aggregate in nitrogen-free NF medium. Red arrows denote microcompartments.

HD03 formed light pink, smooth colonies on M14 agar with ovoid cells (0.5 to 1.0 µm) forming rosette-like aggregates and filamentous appendages—hallmark features of *Planctomycetota* (Fig. 1a and b) (32). Growth kinetics in liquid medium showed that HD03 reached stationary phase at 70 h, with a doubling time of 10.8 h—slower than its closest related strain *C. conspicua* Mal65^T^ (8 h), but faster than other *C. conspicua* strains, such as Pan14r (25 h) and V7 (12 h) (33). Ecophysiological assays revealed exceptional adaptability: HD03 can grow within a temperature range of 10 to 40°C, with an optimal growth temperature of 33°C. It also thrives in a pH range of 5 to 9, with an optimal pH of 7.5. These features exhibited minor, yet distinct, differences when compared to its closely related strain (Table S1). HD03 can utilize a variety of carbon sources, including mannitol, rhamnose, glucose, fucose, laminaran, and fucoidan (Fig. S1). Additionally, HD03 exhibits a broad spectrum of antibiotics, resisting 21 out of 31 tested drugs, including ampicillin, penicillin, and oxacillin (Table S2). This trait aligns with the universal antibiotic resistance profile observed across *Planctomycetota* clades (32).

**Fig 2:**
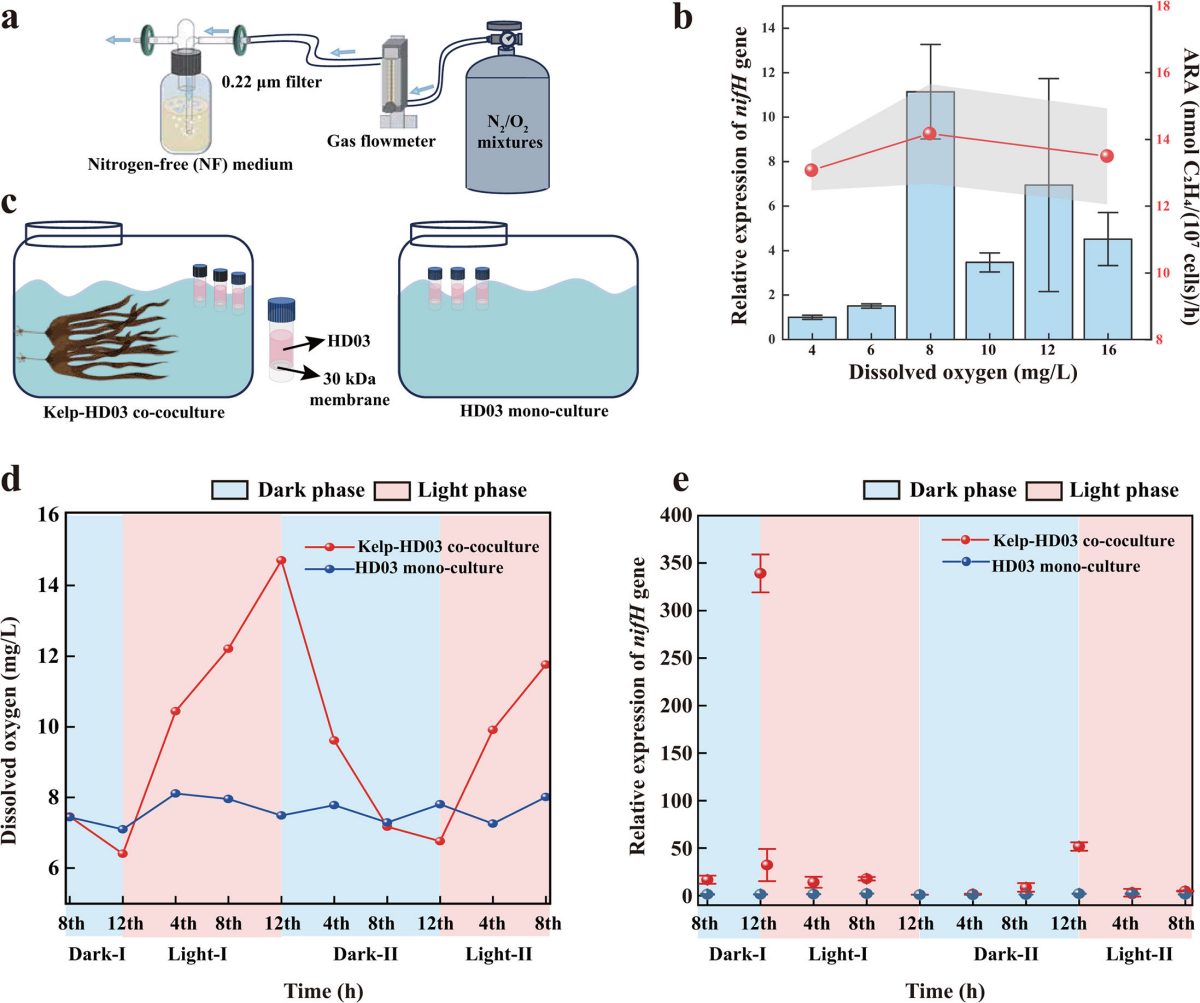
Nitrogen fixation *nifH* gene expression of *Crateriforma* sp. HD03 under different dissolved oxygen concentrations and co-culture conditions with macroalgae. (**a**) Schematic of the aeration apparatus for the oxygen gradient experiment. (**b**) Blue bars represent relative *nifH* expression of HD03 across different dissolved oxygen concentrations. Relative expression was calculated via the ΔΔCt method, normalized to 16S rRNA gene, and calibrated to the 4 mg/L DO condition (set as the control for lowest expression). The red dotted line represents nitrogenase activity measured by the acetylene reduction assay (ARA) with gray shading as error bars. (**c**) Schematic of the kelp-HD03 co-culture and HD03 mono-culture experiments. Healthy kelps were transferred to a sterile bucket with seawater containing F/2 medium. HD03 cells in NF medium were introduced via custom diffusion chambers (downside fitted with bacteria-impermeable filters). Inoculated chambers were immersed in the bucket, with HD03 monoculture controls set up in identical buckets. Triplicate co-cultures and controls were maintained under a 12 h light/dark cycle (30 µmol/m²/s, 15°C). (**d**) Red and blue lines indicate diurnal dissolved oxygen fluctuations in the co-culture and mono-culture systems, respectively. (**e**) Red and blue lines represent diurnal *nifH* expression dynamics in kelp-HD03 co-culture and HD03 mono-culture systems, respectively. Relative expression was calculated via the ΔΔCt method, normalized to the 16S rRNA gene, and calibrated to the expression level at the 12 h light exposure time point for co-culture and mono-culture, respectively. Data are presented as the mean ± standard deviation (SD, *n* = 3).

For functional validation of nitrogen fixation, HD03 grew in nitrogen-free (NF) medium, reaching OD_600_ = 0.8 after 5 days—evidence of atmospheric nitrogen utilization for growth. Notably, HD03 failed to grow anaerobically, confirming its obligate aerobic nitrogen fixation. The acetylene reduction assay (ARA) quantified aerobic nitrogen fixation rate by the strain at 14.2 ± 1.5 nmol C_2_H_4_/(10^7^ cells)/h, determined after 3 days of cultivation in NF medium under 8 mg/L dissolved oxygen (DO), when cell density reached approximately 6 × 10^6^ cells/mL. Cell-free controls showed no activity, confirming that the nitrogen fixation activity was attributable to cellular metabolism. Furthermore, to investigate the oxygen tolerance range of nitrogen fixation, we designed experiments with controlled DO (4–16 mg/L) in cultures of HD03 (Fig. 2a). Under these conditions, HD03 maintained *nifH* gene expression and nitrogenase activity, with ARA measurements ranging from 13.1 ± 0.6 to 14.2 ± 1.5 nmol C_2_H_4_/(10^7^ cells)/h, demonstrating its ability to mitigate oxygen stress during aerobic nitrogen fixation—an exceptional trait among nitrogen-fixing bacteria. These results collectively confirm that HD03 is the first experimentally validated nitrogen fixer in marine *Planctomycetota* in pure culture.

### Molecular and physiological strategies enabling aerobic nitrogen fixation in *Crateriforma* sp. HD03

A striking physiological trait of HD03 is its ability to maintain significant nitrogen fixation activity under high oxygen tension. At DO levels of 16 mg/L, HD03 maintains detectable *nifH* expression and nitrogen fixation rates (13.1 ± 0.6 nmol C_2_H_4_/(10^7^ cells)/h) compared to those at 4 mg/L DO and only slightly lower than the peak rate at 8 mg/L DO (Fig. 2b). This tolerance far exceeds the reported 5.28 mg/L threshold for *Pseudomonas stutzeri* BAL361 (34) and contrasts with most nitrogen-fixing bacteria, which require microoxic or anaerobic niches to protect oxygen-sensitive nitrogenase. Model diazotrophs, such as *Azotobacter vinelandii* and soil-derived *Pseudomonas stutzeri* strains, exhibit lower oxygen tolerance during nitrogen fixation (35–37), underscoring the exceptional aerobic adaptation of HD03.

**Fig 3:**
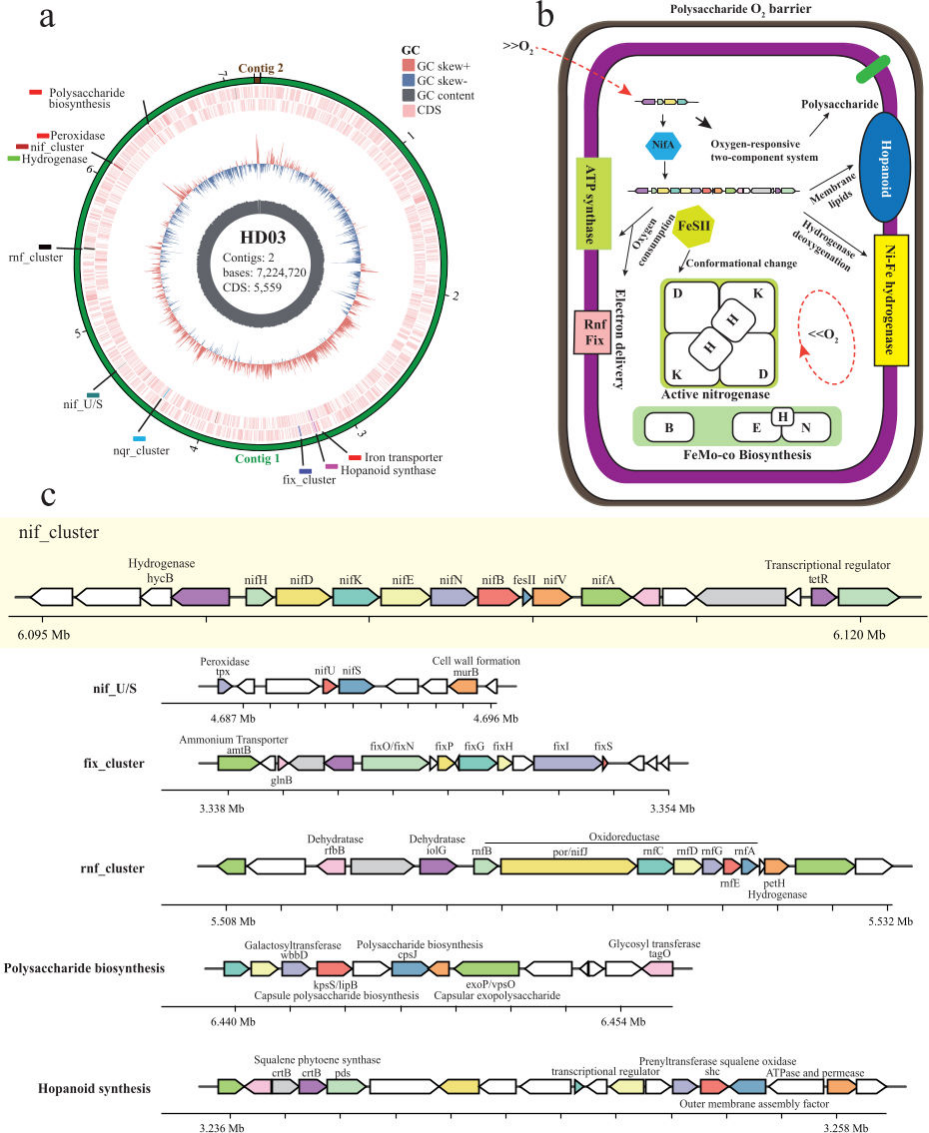
Genomic blueprint unveils aerobic nitrogen fixation adaptations in *Crateriforma* sp. HD03. (**a**) Circular genome of HD03, layered from inner to outer: GC content (gray), GC skew (forward: red; reverse: blue), and coding sequences (CDSs, pink). Outer rings highlight clusters for nitrogen fixation, iron acquisition, oxygen defense, and polysaccharide biosynthesis. (**b**) Schematic of aerobic nitrogen fixation strategy decoded genomics: two-component system-mediated O_2_ sensing, respiratory oxygen consumption, hydrogenase-driven O_2_ scavenging, physical barriers (polysaccharide capsule, hopanoid membranes), and FeSII-dependent nitrogenase shielding. (**c**) Core gene clusters governing nitrogen fixation and metabolism. nif_cluster: encodes nitrogenase, hydrogenase, and FeSII; nif_U/S: regulates *nif* transcription; fix_cluster: controls respiratory O_2_ utilization; rnf_cluster: delivers low-potential electrons to nitrogenase; polysaccharide biosynthesis: constructs protective capsules; hopanoid synthesis: builds hopanoid-rich membranes.

To decipher the molecular basis of this adaptation, we performed hybrid genome sequencing using PromethION long-read and Illumina short-read technologies. The 7.22 Mb genome, assembled into two contigs with 57.75% GC content, encodes 6,543 protein-coding genes annotated across 228 SEED subsystems and 76 RNA genes (Fig. 3a; Table S3). Genomic screening identified a complete *nifHDKBEN* gene cluster, encoding the canonical molybdenum-dependent nitrogenase complex. The structural genes *nifHDK* and maturation genes *nifBEN* are intact, consistent with functional nitrogenase activity (38). Notably, no alternative nitrogenase genes (*vnf*/*anf*) were detected, indicating a strict dependency on the molybdenum-based system, as observed in other aerobic diazotrophs where the *nif* cluster serves as the primary nitrogen-fixation machinery (39). Subsequently, six interconnected genomic modules underpin HD03’s oxygen tolerance during nitrogen fixation were identified (Fig. 3b and c).

- Enzymatic oxygen scavenging and structural protection: The *nif* gene cluster harbors hydrogenase (*hya*) and Shethna protein II (FeSII) genes, forming a dual-defense system. Hydrogenase-mediated H_2_ oxidation consumes intracellular oxygen while generating ATP, establishing a microoxic niche conducive to nitrogenase activity (40). Concurrently, FeSII protein stabilizes the nitrogenase metallocluster via conformational changes, protecting it from oxidative damage (41).
- Transcriptional and oxygen metabolic regulation: The *nifA* gene encodes a transcriptional regulator that modulates nitrogenase expression (38). NifA activity is responsive to oxygen status, with conserved cysteine residues implicated in oxygen sensing (42). The *fixOPGHIS* operon further modulates respiratory oxygen consumption, dynamically maintaining a microoxic environment optimal for nitrogenase activity (37).
- Redox network diversification: The *rnfABCDEG* gene cluster, encoding an electron transfer complex, is associated with three hydrogenase genes (*rfbB*, *iolG*, and *petH*). This redundancy enables flexible electron sourcing from diverse donors (e.g., H₂, algal exudates), supporting nitrogenase activity across oxygen gradients (38).
- Membrane lipid-mediated oxygen barrier: Genes for sterol/hopanoid synthesis (*crtB* and *she*) modify membrane properties. Hopanoids intercalate into phospholipid bilayers to reduce fluidity and oxygen permeability (43), while sterols enhance membrane structural stability. This lipid remodeling creates a physical barrier that segregates nitrogenase within microdomains, minimizing direct oxygen exposure (44).
- Biofilm-driven microenvironment: Genes for polysaccharide biosynthesis (*kps*, *exo*) facilitate aggregate or biofilm formation. Outer-layer cells in these structures consume oxygen via respiration, creating microoxic cores ideal for nitrogen fixation (45). Biofilm attachment to algal surfaces further minimizes oxygen exposure while optimizing nutrient uptake, mirroring strategies in particle-associated marine diazotrophs (46, 47).
- Microcompartmentalization potential: Genes associated with bacterial microcompartments (*ackA*) suggest a mechanism to encapsulate nitrogenase in proteinaceous structures (48). Microscopy revealed microcompartment-like structures under nitrogen limitation (Fig. 1c through f), although their precise role in oxygen protection requires further validation.

Complementing these genomic adaptations, HD03 exhibits distinctive cellular and physiological traits. As a member of *Planctomycetota*, it forms rosette-like aggregates (32, 49) that likely generate internal oxygen gradients, shielding inner cells from oxidative stress (50). Transmission electron microscopy confirmed abundant fibrils and attachment structures, while crystal violet staining validated biofilm formation. These features enhance adherence to algal surfaces and promote microoxic niche development.

Beyond nitrogen fixation, HD03 exhibits versatile nitrogen metabolic capabilities. Genes for ammonia assimilation, nitrate transporters, and nitrate/nitrite ammonification enable the utilization of inorganic nitrogen sources. Additionally, the presence of N-acetylglucosamine transporters, glucosamine-6-phosphate deaminase, and N-acetylglucosamine-6-phosphate deacetylase genes indicates capacity for organic nitrogen utilization. This metabolic flexibility likely allows HD03 to thrive in dynamic marine environments with fluctuating nutrient availability.

### Identification of two distinct nitrogen-fixing clades of *Planctomycetota* in pure culture

**Fig 4:**
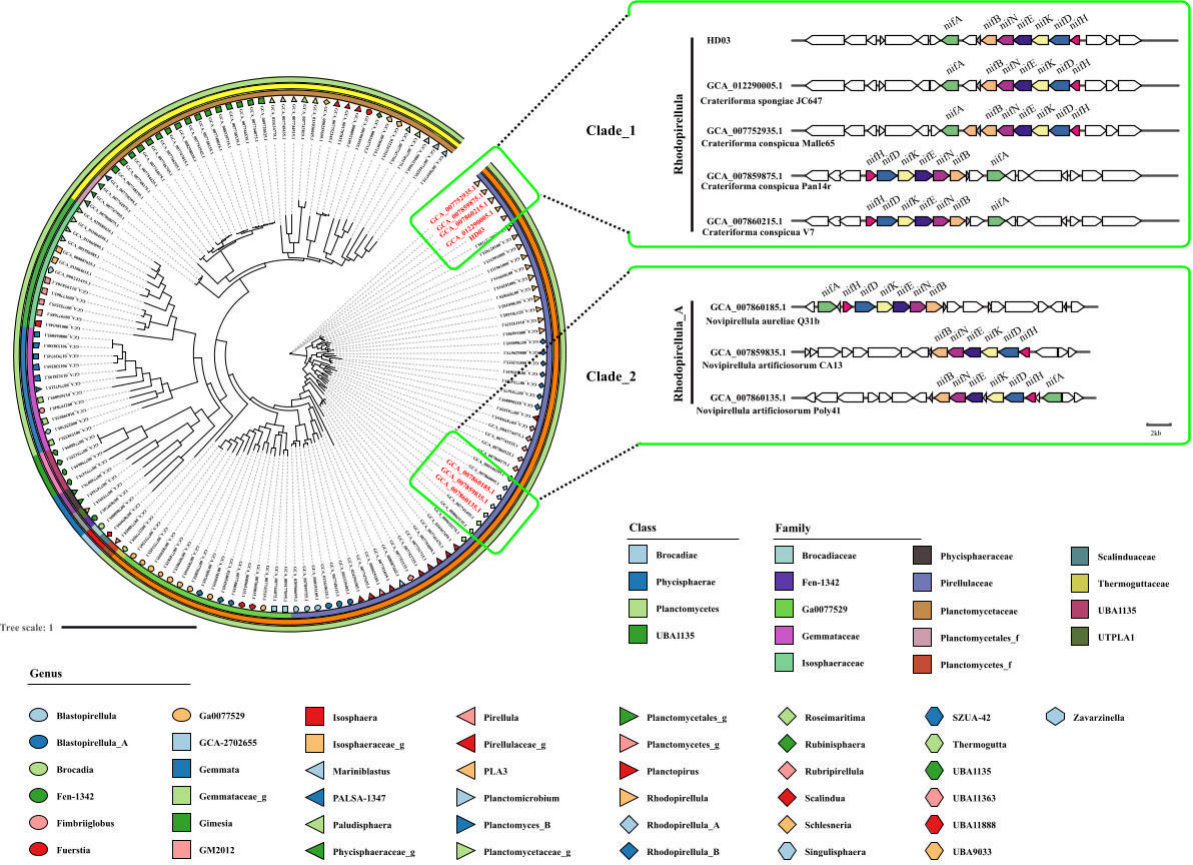
Phylogenetic tree of *Planctomycetota* genomes revealing nitrogen fixation gene cluster distribution. The phylogenetic tree was constructed for 142 *Planctomycetota* genomes retrieved from NCBI public databases and *Crateriforma* sp. HD03. Using PhyloPhlAn 3.0 with 400 universal marker genes, the tree’s circular layers denote taxonomic ranks (outer to inner: class, order, family, and genus). Eight genomes harbor complete nitrogen fixation core gene clusters, segregating into two clades (Clade 1: *Rhodopiellula*; Clade 2: *Rhodopiellula*_A). Upper-right insets detail these strains and their nitrogen fixation gene clusters.

To determine the prevalence and extent of nitrogen fixation potential among pure-cultured *Planctomycetota* strains, we conducted an extensive genomic survey. By scrutinizing 142 strains sourced from public databases, with a particular focus on the presence of intact *nifHDK* core gene clusters—crucial genetic determinants of functional nitrogenase complexes—we successfully identified two distinct clades of *Planctomycetota* equipped with complete nitrogen fixation capabilities (Fig. 4).

The *Rhodopirellula* clade comprises five strains, including *Crateriforma conspicua* Mal65^T^ and HD03, all classified under the genus *Crateriforma*. These strains are characterized by their ability to form biofilms and display rosette-like aggregation patterns. Such traits play a vital role in alleviating oxygen stress in aerobic conditions. The *Rhodopirellula*_A clade encompasses three strains belonging to the genus *Novipirellula* (e.g., *Novipirellula artificiosorum* CA13). Although genetically distinct from the *Crateriforma* strains, they share conserved *nifHDK* clusters.

Notably, global metagenomic data from the Tara Oceans expedition brought to light a third, uncultivated clade: *Phycisphaeraceae*_UBA7800. This clade dominates nitrogen-fixing communities associated with 0.22–1.6/3 µm particles in the surface oceans (19, 51). Phylogenetic analysis demonstrates that the two cultivable clades, *Crateriforma* and *Novipirellula*, are separate lineages, distinct from the uncultivated *Phycisphaeraceae* clade. This divergence highlights the varied distribution and evolutionary paths of nitrogen fixation within *Planctomycetota*. While functional validation of nitrogen fixation in these lineages is pending, the experimentally established diazotrophy of HD03 provides inferential support for nitrogen-fixing capacity in these clades.

### Circadian rhythm of nitrogen fixation in *Planctomycetota* within algal environments

A notable finding is that many above-mentioned culturable nitrogen-fixing *Planctomycetota* strains originate from macroalgal environments. For instance, strains *C. conspicua* Mal65^T^ and *N. artificiosorum* CA13 were isolated from the surfaces of macroalgae, and the *Phycisphaeraceae*_UBA7800 clade identified in the Tara Oceans expedition shows a preference for particle sizes consistent with those of phytoplankton (19, 51). These findings strongly imply that algal environments may represent a crucial ecological niche for nitrogen-fixing *Planctomycetota*. This ecological association likely underpins a mutualistic relationship. *Planctomycetota* convert atmospheric nitrogen into bioavailable forms, providing vital nutrients for algae. In return, algae release organic exudates that serve as carbon and energy substrates for *Planctomycetota*. Moreover, the biofilms formed by *Planctomycetota* on algal surfaces create microoxic microhabitats, shielding oxygen-sensitive nitrogenase enzymes from inactivation.

Considering that the diurnal photosynthetic cycle of algae modulates oxygen and nutrient dynamics, we investigated the 24-hour *nifH* gene expression process of HD03 within algal environments. A co-culture system of strain HD03 and kelp (*Saccharina japonica*) was established. The system was subjected to a 12 h light/dark cycle, emulating natural photoperiodic conditions. Oxygen profiling revealed distinct diurnal fluctuations: during the dark phase, oxygen concentrations decreased to 6.7 mg/L, creating microoxic conditions conducive to nitrogenase activity, whereas photosynthetic activity during the light phase elevated oxygen levels to 14 mg/L, indicative of fully aerobic conditions (Fig. 2d). Significantly, relative expression of the *nifH* gene was exclusively detected during the dark phase (Fig. 2e), coinciding with reduced oxygen stress. In contrast, in monocultures of HD03 without kelp, no obvious regular fluctuations were observed in DO and relative *nifH* expression (Fig. 2e). This circadian pattern of gene expression represents an additional mechanism by which *Planctomycetota* synchronize nitrogen fixation with algal photosynthesis, analogous to the temporal separation strategies documented in cyanobacteria (30, 31). Additionally, HD03 exhibited elevated nitrogenase activity at the dark-light interface, consistent with observations that nitrogenase activity in hot spring microbial mats peaks in the evening and early morning (52). Notably, although strain HD03 possesses the capacity for oxygen-tolerant nitrogen fixation, it primarily carries out this process during low-oxygen nighttime periods. This seemingly contradictory behavior likely represents an adaptation to its algal habitat. First, this may be attributed to the strain’s energy allocation requirements: although HD03 can fix nitrogen under high-oxygen conditions, the regulatory process for nitrogen fixation under oxygen stress is empirically energy-intensive. Temporal segregation of nitrogen fixation minimizes inhibition of nitrogenase by oxygen and conserves cellular energy, balancing tolerance with metabolic efficiency. Furthermore, the complex impacts of daytime conditions on nitrogen fixation should be noted. Algal photosynthesis introduces complex factors affecting nitrogen fixation, such as the photosynthetic metabolites. These substances, in conjunction with photosynthetic oxygen production, may exert complex regulatory effects on nitrogen fixation in *Planctomycetota*, which warrants further in-depth investigation. It should be acknowledged that the excess nitrate added to sustain algae in co-cultures may have influenced HD03’s metabolism, suggesting that the interaction between algae and nitrogen-fixing bacteria may follow different patterns under oligotrophic conditions. Future environmental surveys should prioritize the nighttime sampling of metatranscriptomes from nitrogen-fixing microorganisms, along with measurements of nitrogen fixation rates, to capture circadian dynamics.

In summary, the characterization of nitrogen fixation in strain HD03, combined with the widespread distribution of *nifHDK* gene clusters in *Planctomycetota*, firmly establishes this phylum as a previously underappreciated keystone in marine nitrogen input. Future research should focus on functional validation of uncultivated *Planctomycetota* clades and mechanistic studies of oxygen tolerance, which may provide insights for enhancing nitrogen supply in nutrient-limited marine ecosystems.

## MATERIALS AND METHODS

### Isolation of *Planctomycetota* strains from algal environments

Considering the potential occurrence of nitrogen-fixing *Planctomycetota* in the macroalgal habitats, we targeted the brown alga *Saccharina japonica* collected from fresh thalli in nearshore ecosystems for strain isolation. Macroalgal samples were repeatedly rinsed with sterile seawater to remove surface planktonic microorganisms, then diced into small fragments and vortexed with sterile seawater to generate bacterial suspensions. Serial dilutions of the suspensions were evenly spread on M14 agar plates supplemented with 200 mg/L ampicillin, 1,000 mg/L streptomycin, and 20 mg/L cycloheximide, which selectively enriched for *Planctomycetota* while inhibiting fungal contamination (13). The plates were incubated in the dark at 28°C for two weeks until visible colony formation occurred. Colonies morphologically consistent with *Planctomycetota* (e.g., pink-tinged colonies) were selected and purified through at least three successive streaking rounds on agar plates (12). Growth rates and doubling times were determined from the slopes of ln (OD_600_) versus cultivation time plots, based on three biological replicates cultured in M14 medium. The 16S rRNA gene of purified strains was amplified via PCR, and products were sequenced at Sangon Biotech Co., Ltd. (Qingdao, China). The 16S rRNA gene sequences were taxonomically classified using the EZBioCloud platform to determine strain identities (53). Additionally, strains were screened for nitrogen-fixing genes using *nifH* gene primers POLF (5′-TGCGAYCCSAARGCBGACTC-3′) and POLR (5′-ATSGCCATCATYTCRCCGGA-3′) (54). Through this workflow, *Crateriforma* sp. HD03, a *Planctomycetota* strain harboring the nitrogen-fixing *nifH* gene, was identified from the surface of *Saccharina japonica*.

### Evaluation of growth characteristics and physiological properties of *Crateriforma* sp. HD03

The optimal growth conditions and tolerance limits were assessed in M14 medium (13). To ascertain the optimal growth temperature, the strain was cultured at 10, 22, 24, 29, 33, 40, and 45°C; growth at 4°C was also examined on M14 agar plates over a one-month period. For pH tolerance testing, the strain was cultured in 24-well plates containing 10 mM buffer systems: HAc/NaAc (pH 4.5), MES (pH 5.5), PIPES (pH 6.5), HEPES (pH 7.5), and CAPS (pH 8.5 and 9.5) (55). Anaerobic growth was evaluated by incubating the strain in an anaerobic chamber with modified M14 agar medium (supplemented with 0.1% NaNO_3_) at 28°C for 8 weeks.

To establish a carbon-source-specific, nitrogen-free medium for nitrogen-fixation verification, sole carbon source assays were conducted. The strain was cultured in carbon-free medium supplemented with individual carbon sources (1% g/L): mannitol, rhamnose, glucose, fucose, laminaran, and fucoidan. Growth was monitored by measuring OD_600_, with each experiment conducted in triplicate.

Catalase and oxidase activities were assessed using 30% hydrogen peroxide and 1% N,N,N′,N′-tetramethyl-p-phenylenediamine dihydrochloride, respectively. Morphological characteristics were evaluated using light microscopy and transmission electron microscopy (TEM) on cells in the exponential phase. Cells were deposited onto 200-mesh carbon-coated copper grids, negatively stained with 2% uranyl acetate for 3 min, and imaged using an H-7650 TEM. To visualize intracellular microcompartments, strains cultured in nitrogen-containing (M14) or NF media were processed for TEM. Samples underwent sequential infiltration with acetone-embedding agent mixtures, pure embedding agent treatment, polymerization at a defined temperature, ultramicrotomy, and dual staining with lead citrate and uranyl acetate before microscopic observation. NF medium consisted of 0.2 g/L KH₂PO₄, 5 g/L MgSO₄, 0.1 g/L CaSO₄, 10 g/L mannitol, 1 mL/L vitamin solution (56), and 1 mL/L trace-element solution (56), prepared in artificial seawater without nitrogen supplementation.

Biofilm formation capacity was evaluated by culturing the strain in 48-well plates with 0.02% crystal violet. Following 15 min of static incubation, excess dye was removed by washing with sterile water, and biofilm formation was visualized macroscopically (50).

### Assessment of bacterial nitrogen fixation capacity

To evaluate the nitrogen fixation capacity, the strain was pre-cultured in M14 medium for 3 days. Cells were then harvested by centrifugation and resuspended in fresh NF medium to an OD_600_ of 0.8–1.0. Aliquots were transferred to 250 mL sterile bottles containing 50 mL of NF medium, connected to a mass flow controller system (0.5 Pa L/min) delivering nitrogen-oxygen mixtures (Fig. 2a). DO was measured using a Multi3630 IDS analyzer (WTW, Germany), establishing gradients of 4, 8, and 16 mg/L. After three-day cultivation, 20 mL of each culture was transferred to 100 mL sterile anaerobic bottles. Bottles were aerated for 5 min with nitrogen-oxygen mixtures, matching the target DO levels using a sterile gas dispersion tube. Bottles were sealed with isobutyl rubber stoppers and then secured with aluminum caps. For the ARA, 5 mL of headspace gas was withdrawn using a syringe and replaced with an equal volume of acetylene. The sample was incubated at 28°C for 48 h, after which 500 µL of headspace gas was extracted for ethylene quantification using GC-FID (Agilent 7890). Phosphate-buffered saline (PBS) without bacterial cells served as a negative control and was subjected to identical treatment. Ethylene production was normalized to cell density, determined using Accuri II flow cytometer (BD Biosciences, USA) with SYBR Green I staining (57), and nitrogen fixation rates were expressed as nmol C_2_H_4_/(10⁷ cells)/h.

For gene expression analysis, nitrogenase reductase gene *nifH* expression was quantified. Total RNA was extracted using the Vazyme Bacteria RNA Extraction Kit, followed by cDNA synthesis with the Vazyme Hiscript III RT SuperMix for qPCR (+gDNA wiper) kit. gDNA wiper Mix is used to thoroughly remove residual genomic DNA in the RNA template. Quantitative PCR (qPCR) was performed with custom-designed primers: HD3NF (5′-AAAGCACCACCACAGAACAC-3′) and HD3NR (5′-AGCCAGACCGTTCAACAACAATC-3′) for *nifH*, and 16 S-F (5′-TGGCGAAGGCGGCTCAC-3′) and 16 S-R (5′-ATTCATCGTTTACGGTGTGGACTAC-3′) for the 16S rRNA endogenous reference (Sangon Biotech). Reactions were set up using the Vazyme Tap Pro Universal SYBR qPCR Master Mix with cycling conditions: 95°C for 30 s, followed by 40 cycles of 95°C for 10 s and 60°C for 30 s, ending with a melting curve analysis (95°C for 15 s, 60°C for 60 s, 95°C for 15 s). Data were acquired on a LightCycler R480, and relative expression levels were calculated using the ΔΔCt method, normalized to 16S rRNA gene, and calibrated to the 4 mg/L DO condition.

### Genome sequencing, annotation, and functional analysis of *Crateriforma* sp. HD03

HD03 was cultured in M14 liquid medium until reaching OD600 = 0.8. Cells were harvested by centrifugation (12,000 rpm, 10 min), and genomic DNA was extracted using an SDS-based method. DNA quality was assessed by 1% agarose gel electrophoresis, and concentration was quantified using a Qubit 3.0 Fluorometer (Life Technologies, Carlsbad, CA, USA). Genomic DNA was sequenced using a combination of long-read (PromethION) and short-read (Illumina NovaSeq 6000) technologies at Novogene Bioinformatics Technology Co., Ltd. Sequencing data were assembled using the Unicycler pipeline (version 0.5.0) (58), integrating SPAdes, to generate a high-quality genome under default settings. Gene was predicted using Prokka v1.14.6 (59). Gene function was annotated using eggnog-mapper (60) with default parameters. Additionally, the assembled genome was uploaded to the Rapid Annotation using Subsystem Technology (RAST) platform (61) (https://rast.nmpdr.org/rast.cgi) to generate the high-quality functional annotations by conducting a BLAST search against the SEED database (62). The circular genome map and *nif* gene clusters were visualized using the Chiplot platform (https://www.chiplot.online/) (63).

### Phylogenomic analysis of nitrogen-fixing *Planctomycetota*

To investigate the ubiquity and taxonomic distribution of nitrogen-fixing strains in *Planctomycetota*, we compiled 142 genome sequences of *Planctomycetota* strains from NCBI, combined with HD03, yielding a data set of 143 genomes (Table S4). A phylogenomic tree was reconstructed using PhyloPhlAn 3.0 (64), with its default database of 400 universal marker genes, applying the diversity medium parameter to resolve genus- and family-level relationships. The tree was visualized using the Interactive Tree of Life (iTOL) tool (65). Additionally, each genome was screened for *nifHDK* gene clusters using MicrobeAnnotator 2.0.5 (66), with positive detections recorded for phylogenetic correlation. Phylogenetic trees and gene cluster distributions were co-visualized via the Chiplot platform (63).

### Changes in the nifH gene expression of *Crateriforma* sp. HD03 under different oxygen concentrations

To investigate the oxygen sensitivity of nitrogen fixation in HD03, we established a DO gradient using a mass flow controller system delivering nitrogen-oxygen mixtures to 250 mL bottles containing 100 mL of NF medium. DO levels were calibrated to 4, 8, 10, 12, and 16 mg/L (measured by a Multi 3630 IDS analyzer, WTW, Germany). A zero-oxygen condition was excluded due to the strain’s obligate aerobic nature. Bacterial cells were harvested at mid-exponential phase from M14 pre-cultures, washed twice with NF medium, and inoculated into experimental bottles. Cultures were incubated at 28°C for 7 days, maintaining continuous gas flow through sterile 0.22 µm membrane filters (Millipore) fitted at both inlet and outlet ports. Following incubation, triplicate samples were collected, and total RNA was extracted using the Vazyme Bacteria RNA Extraction Kit, followed by cDNA synthesis with the Vazyme Hiscript III RT SuperMix for qPCR (+gDNA wiper) Kit. Expression level of the *nifH* gene was measured by qPCR, as detailed above. For ARA, parallel cultures at 4, 8, and 16 mg/L DO were transferred to 100 mL anaerobic serum bottles. Bottles were sealed with butyl rubber stoppers and aluminum crimp caps, and subsequent acetylene injection, incubation, and gas chromatography analysis were performed as previously described.

### Establishment of macroalgae-HD03 co-culture systems and diurnal detection of nitrogen-fixing gene expression

To characterize nitrogen fixation in HD03 within a macroalgal environment, we established a co-culture system between HD03 and the kelp *Saccharina japonica*. Healthy kelps were collected from the Yellow Sea, China (122.5970 E, 37.2657 N), and rinsed thrice with sterile filtered seawater. These algae were transferred to transparent sterile buckets containing sterile seawater supplemented with F/2 medium (Fig. 2c). After 24 h of acclimation under 12 h light/12 h dark photoperiod (30 µmol/m^2^/s, 15°C) (67), the HD03 cell suspension grown in nitrogen-free (NF) medium for 3 days was introduced using custom-made diffusion chambers. These chambers (50 mL sterile tubes) featured a 30 kDa filter sealed across the bottom, enabling metabolite exchange while preventing bacterial contamination. Chambers were inoculated with 15 mL of HD03 (OD_600_ = 0.8) in NF medium, capped, and immersed in the bucket. Parallel control cultures containing HD03 alone (without kelp) were established in identical buckets with sterile F/2 seawater (Fig. 2c). Triplicate co-cultures and controls were maintained in a temperature-controlled incubator under identical photoperiod conditions.

For diurnal sampling, an 8-hour dark adaptation was followed by harvesting every 4 h. At each time point, HD03 cells were collected by centrifugation, and total RNA was extracted using the Vazyme Bacteria RNA Extraction Kit. The *nifH* expression was quantified by qPCR as previously described. Relative expression levels were calculated using the ΔΔCt method, normalized to the 16S rRNA gene, and calibrated to the minimum expression level observed at the 12 h light exposure time point. Concurrently, DO was measured by a Multi3630 IDS analyzer (WTW, Germany).

## Data Availability

The sequence data for *Crateriforma* sp. HD03 were deposited in GenBank under accession no. CP178615 and CP178616. Strain HD03 was deposited in two publicly accessible culture collections: the Marine Culture Collection of China (MCCC 1K10180) and the China General Microbiological Culture Collection Center (CGMCC 1.65559).

## SUPPLEMENTAL MATERIAL

File S1 aem.01316-25-s0001.pdf Figure S1 and Tables S1 and S2.

File S2 aem.01316-25-s0002.xlsx Tables S3 and S4.

ASM does not own the copyrights to Supplemental Material that may be linked to, or accessed through, an article. The authors have granted ASM a non-exclusive, world-wide license to publish the Supplemental Material files. Please contact the corresponding author directly for reuse.
