# Supplementary material for: Oxygen-tolerant nitrogen fixation in a marine alga-colonizing Planctomycetota
Source: Appl Environ Microbiol. 2025 Oct 20;91(11):e01316-25. doi: 10.1128/aem.01316-25 (PMC12628686; doi:10.1128/aem.01316-25)
Supplement: File S1 — Figure S1 and Tables S1 and S2. [file aem.01316-25-s0001.pdf]

Oxygen-tolerant nitrogen fixation in a marine alga-colonizing *Planctomycetota*

Zenghu Zhang[2]†, Ziwei Wang[1,2]†, Peichen Teng[2], Tong Yu[1], Yongyu Zhang[2,3,4]*

[1] School of Environmental and Municipal Engineering, Qingdao University of Technology, Qingdao, 266520, China

[2] Qingdao New Energy Shandong Laboratory, Qingdao Institute of Bioenergy and Bioprocess Technology, Chinese Academy of Sciences, Qingdao, 266101, China

[3] Southern Marine Science and Engineering Guangdong Laboratory (Zhuhai), Zhuhai 519000, China

[4] Laboratory for Marine Biology and Biotechnology, Qingdao Marine Science and Technology Center, Qingdao, 266237, China

***Correspondence**:

Yongyu Zhang, zhangyy@qibebt.ac.cn

† Zenghu Zhang and Ziwei Wang contributed equally to this work.

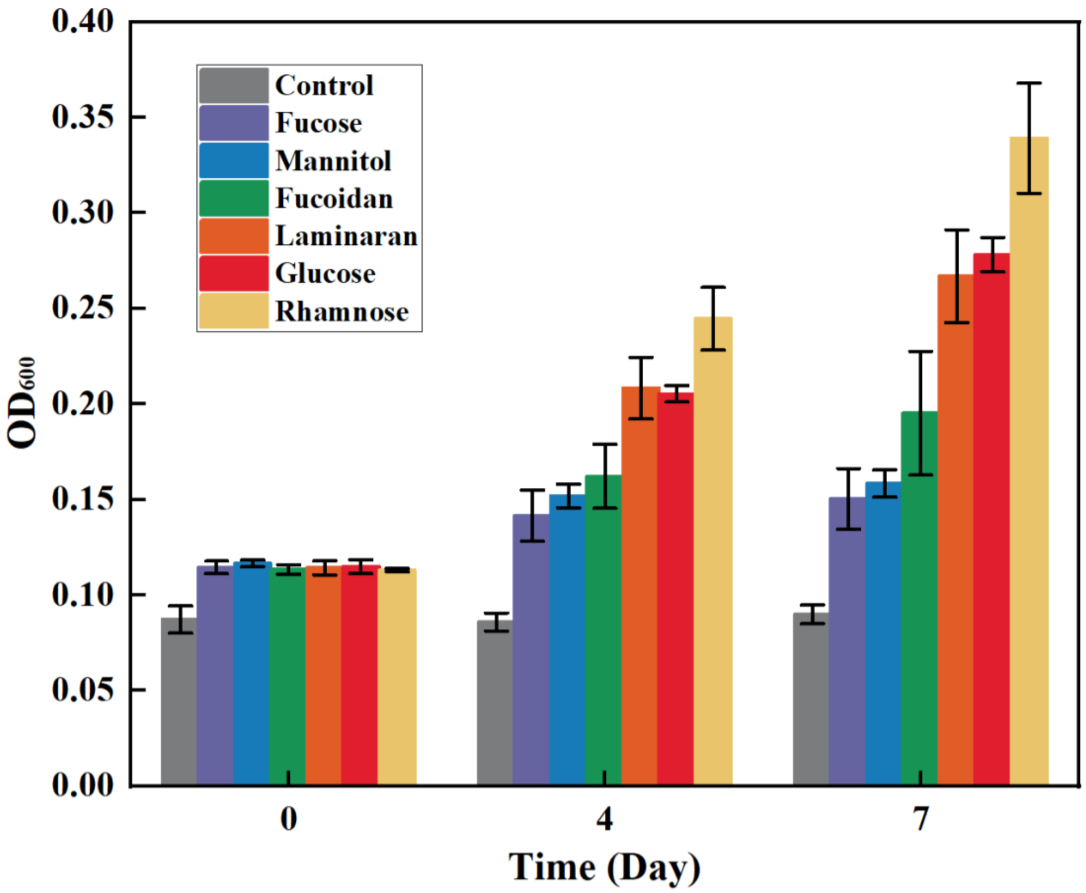

Figure S1: **Sole carbon source utilization of *Crateriforma* sp. HD03.** The strain was cultured in carbon-free medium supplemented with individual carbon sources (1% g/L): mannitol, rhamnose, glucose, fucose, laminaran, and fucoidan. Growth was monitored by measuring OD_{600}, with each experiment conducted in triplicate.

Table S1: Genetic and physiological differences between *Crateriforma* sp. HD03 and closely related strains

Characteristics	HD03	Mal65#	Pan14r#	V7#
**Doubling time (h)**	10.8	8	25	12
**Growth at/in**				
Temperature °C (optimum)	10–40 (33)	10–36 (36)	No available	No available
pH (optimum)	5.0–9.0 (7.5)	5.0–9.5 (7.5)	No available	No available
**Genomic information**				
Genome size (bp)	7,224,720	7,182,433	7,137,949	7,227,508
GC (%)	57.7± 0	57.8 ± 0	57.8 ± 2.5	57.7 ± 2.7
Completeness (%)	100.00	95.69	94.83	94.83
Protein-coding genes	5650	5437	5400	5531
**The number of *nif* genes***				
nifD nifK nifE nifN	4	2	2	3
nifH	1	1	1	1

* Note: Gene annotation was performed using microbeannotator-2.0.5 (Ruiz-Perez et al., 2021).

# Note: Data for these strains were obtained from Peeters et al. 2020.

Ruiz-Perez, C.A., Conrad, R.E. & Konstantinidis, K.T. MicrobeAnnotator: a user-friendly, comprehensive functional annotation pipeline for microbial genomes. BMC Bioinformatics 22, 11 (2021). https://doi.org/10.1186/s12859-020-03940-5

Peeters, S.H., Wiegand, S., Kallscheuer, N. et al. Three marine strains constitute the novel genus and species *Crateriforma conspicua* in the phylum *Planctomycetes*. Antonie van Leeuwenhoek 113, 1797–1809 (2020). https://doi.org/10.1007/s10482-019-01375-4

Table S2 The antibiotic resistance spectrum of *Crateriforma* sp. HD03

Antibiotics	Resistance		Antibiotics	Resistance
Penicillin	√		Tetracycline	×
Oxacillin	√		Doxycycline	×
Ampicillin	√		Minocycline	√
Carbenicillin	√		Erythromycin	×
Piperacillin	√		Midecamycin	×
Cephalexin	√		Norfloxacin	×
Cefazolin	√		Ofloxacin	×
Cefradine	√		Ciprofloxacin	×
Cefuroxime	√		Vancomycin	√
Ceftazidime	√		Polymyxin B	×
Ceftriaxone	√		Sulfamethoxazole	√
Cefoperazone	√		Furazolidone	√
Amikacin	√		Chloramphenicol	×

Gentamicin	√	Clindamycin	√
Kanamycin	√	Cefuroxime	×
Neomycin	√		

Table S3: The list of functional genes in the strain *Crateriforma* sp. HD03 genome

The table is detailed in the Excel file in the attachment.

Table S4 List and taxonomic status of 143 genomes: 142 *Planctomycetota* strains from NCBI and *Crateriforma* sp. HD03

The table is detailed in the Excel file in the attachment.
